# Early neuroimaging markers of *FOXP2* intragenic deletion

**DOI:** 10.1038/srep35192

**Published:** 2016-10-13

**Authors:** Frédérique J. Liégeois, Michael S. Hildebrand, Alexandra Bonthrone, Samantha J. Turner, Ingrid E. Scheffer, Melanie Bahlo, Alan Connelly, Angela T. Morgan

**Affiliations:** 1 UCL Institute of Child Health, London, UK; 2 Great Ormond Street Hospital for Children NHS Trust, London, UK; 3 University of Melbourne, Australia; 4 Florey Institute of Neuroscience and Mental Health, Melbourne, Australia; 5 Austin Health, Melbourne, Australia; 6 Murdoch Childrens Research Institute, Melbourne, Australia; 7 The Walter and Eliza Hall Institute of Medical Research, Melbourne, Australia; 8 Royal Children’s Hospital, Melbourne, Australia

## Abstract

*FOXP2* is the major gene associated with severe, persistent, developmental speech and language disorders. While studies in the original family in which a *FOXP2* mutation was found showed volume reduction and reduced activation in core language and speech networks, there have been no imaging studies of different *FOXP2* mutations. We conducted a multimodal MRI study in an eight-year-old boy (A-II) with a *de novo FOXP2* intragenic deletion. A-II showed marked bilateral volume reductions in the hippocampus, thalamus, globus pallidus, and caudate nucleus compared with 26 control males (effect sizes from −1 to −3). He showed no detectable functional MRI activity when repeating nonsense words. The hippocampus is implicated for the first time in *FOXP2* diseases. We conclude that *FOXP2* anomaly is either directly or indirectly associated with atypical development of widespread subcortical networks early in life.

*FOXP2*^1^ is the only identified gene associated with a severe, and persistent developmental speech and language disorder in the setting of preserved cognition. Its discovery through the study of a multigenerational British family (the KE family^2^) has allowed scientists to explore the possible molecular pathways associated with dysfunction of speech and language networks in the brain^3^,^4^. This body of work has also informed the possible mechanisms linked to the emergence of speech and language skills during human evolution^5^,^6^. About 20 individuals with either *de novo* or familial *FOXP2* disruptions that only affect the transcript of *FOXP2*, and no other surrounding genes, have been reported worldwide. All affected individuals share a common phenotype of childhood apraxia of speech, oral dyspraxia and language impairment^7^. Most cases present with normal brain MRIs, suggesting that this severe phenotype arises from subtle functional brain anomalies. No advanced brain imaging data have been reported on children with *FOXP2* mutations. Studies have focused on imaging adult members of the KE family^2^,^8^,^9^,^10^, thereby imaging them decades after the emergence of their speech-language impairment. As a result, some of these family-specific findings could be due to compensation strategies, neural reorganization, or both. Altogether, there is a lack of data on how *FOXP2* abnormality disrupts human development, affecting brain structure and function in childhood, and the development of white matter connectivity crucial for speech and language.

Here we report multimodal neuroimaging findings in an eight-year-old boy with a unique *de novo* intragenic *FOXP2* deletion, case A-II^7^, whose speech and language profile is consistent with the previously described *FOXP2* phenotype. We aimed to identify early markers of brain dysfunction by combining functional and structural MRI methods. We used whole-brain as well as *a priori* defined regions of interest analyses and compared A-II’s data to those of age-matched typically developing males. Based on neuroimaging findings from the affected members of the KE family, we hypothesized that A-II would show bilateral grey matter reductions in the head of the caudate nucleus, precentral gyrus, cerebellum, and dorsal inferior frontal gyrus^8^,^11^. Grey matter increases were predicted in the angular gyrus and posterior superior temporal gyrus^11^. We also predicted fMRI underactivity during nonsense word repetition (a phenotypic marker^8^,^12^) in the speech related sensorimotor cortex bilaterally, the left rolandic operculum, the left putamen, and Broca’s area (pars opercularis and triangularis)^9^,^10^. Finally, we hypothesized atypical microstructure of the arcuate fasciculus/superior longitudinal fasciculus, two white matter tracts involved in the “dorsal” language stream responsible for the transformation of speech sounds into articulatory productions^13^,^14^.

## Results

### Global brain measures are preserved

Whole brain grey matter (mean = 0.857l, 38th percentile; Effect size for the difference = −0.299, 95% CI −0.689 to 0.097) and white matter (mean = 0.389l; Effect size for the difference = −1.268, 95% CI −1.780 to −0.741) volumes for case A-II were within the normal range relative to the control group (mean for grey = 0.877, SD = 0.067; mean for white = 0.460l, SD = 0.056).

### Whole-brain method reveals grey matter anomalies in the hippocampus and caudate nucleus

**Figure 1:**
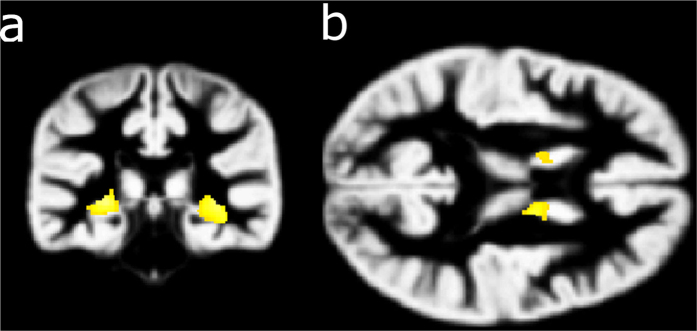
Bilateral grey matter reductions in A-II relative to the control group. Reductions in the hippocampus (**a**) and the head of the caudate nucleus (**b**) were revealed using VBM after correcting for age and total brain grey matter volume. Results are displayed at p = 0.001, uncorrected for multiple comparisons and superimposed onto the study-specific grey matter template. Left hippocampus: peak at 33, −26, −3; cluster size = 738 voxels, T = 5.77, p = 0.046 FWE; Right hippocampus: peak at −26, −30, −2, cluster size = 324 voxels; T = 4.04, p < 0.001 uncorrected; Left caudate head: peak at −14, −3, 18, cluster size = 46 voxels; T = 3.61, p < 0.001 uncorrected; Right caudate head: peak at 14, −6, 16, cluster size = 158 voxels; T = 4.22, p < 0.001 uncorrected.

Voxel Based Morphometry (VBM) revealed bilateral reductions (see Fig. 1) in a large cluster within the hippocampus (left peak, cluster size 738 voxels, p = 0.046, FWE correction; right peak, cluster size 324 voxels, p < 0.001 uncorrected) and in the head of caudate nucleus (p < 0.001 uncorrected, but *a priori* hypothesized). One voxel (peak at 27, 3, −2) within the right putamen was significant at uncorrected level (T = 2.68, p = 0.007, cluster size 61 voxels) but did not survive small volume correction. No bilateral increases were detected at p = 0.05 (Family Wise Error correction or FWE). No significant bilateral reductions or increases were found in any of the 25 control participants when compared to the remainder of the control group.

### Regional volumetric analysis reveals reductions in the hippocampus and basal ganglia

**Figure 2:**
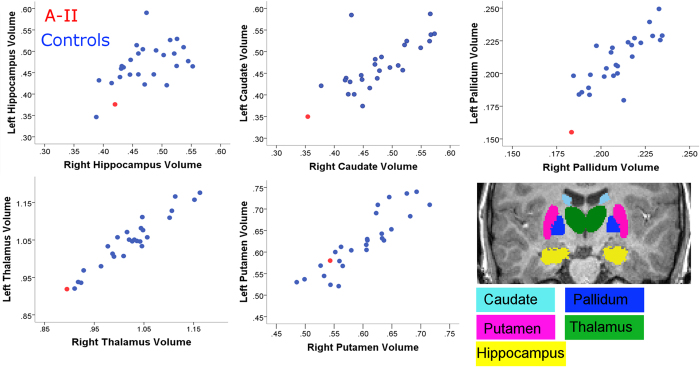
Left and right regional volumes in A-II (red circle) relative to age-matched control males (blue circles). Volumes are expressed in percentages of total brain grey matter volumes. Insert, example of segmented regions of interest from a control participant (coronal view).

Bilateral reductions beyond one standard deviation were detected in all regions of interest except the putamen (see Table 1 and Fig. 2). For the globus pallidus and hippocampus, reductions were only statistically significant in the left hemisphere. Values survived correction for multiple comparison in the globus pallidus only. We did not detect any statistically significant volume increases in A-II relative to the control group in any of these *a-priori* regions of interest.

### White matter anomalies in speech and language related tracts

No fractional anisotropy (FA) differences between A-II and the control group were statistically significant (Table 2). FA reductions above one standard deviation were however detected for the right dorsal corticobulbar tract, the left arcuate fasciculus, left superior longitudinal fasciculus and left cingulum bundle. FA increases were detected in the right arcuate fasciculus.

### fMRI activation during nonsense word repetition

**Figure 3:**
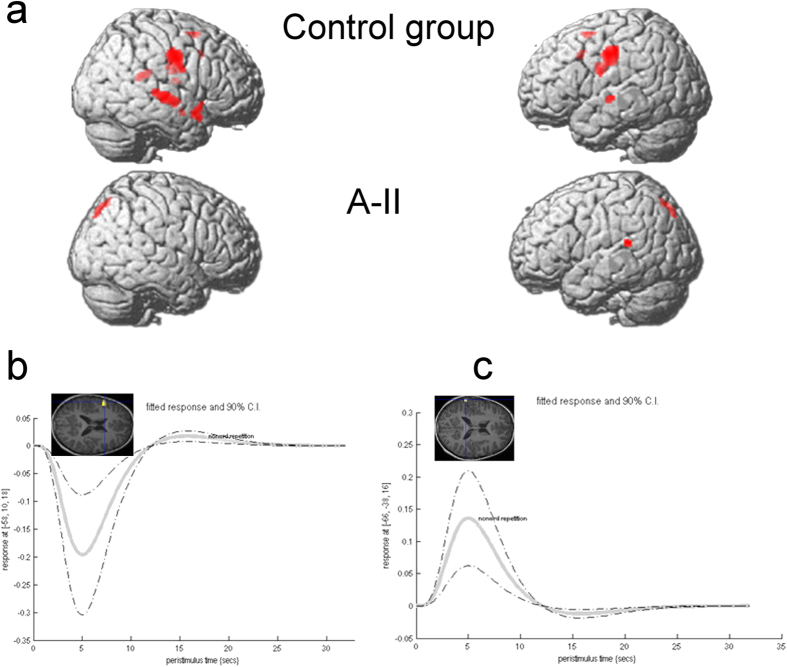
fMRI activation during nonsense word repetition. (**a**) Average activation in the control group (top row) and in A-II (bottom row). Results are displayed at an intensity threshold of p = 0.05 family-wise error correction for the group (minimum cluster size 10 voxels) and p = 0.005 uncorrected for multiple comparisons for A-II, and projected onto a single brain rendering. (**b**) Signal change in the left inferior frontal gyrus peak (insert) shows negative BOLD response in A-II. (**c**) Signal change in the superior temporal gyrus peak (insert) shows positive BOLD response in A-II.

A-II showed no supra-threshold detectable activation during the Nonword repetition vs. Baseline contrast, despite performing the task (Fig. 3). Activation was detected at p = 0.005, uncorrected for multiple comparisons in the left posterior superior temporal gyrus (hypothesized, peak at −66, −38, 16; T = 3.04, p = 0.002, cluster size 17 voxels; see Fig. 2a). The control group showed extensive activation involving the bilateral sensorimotor and superior temporal cortices, as well as the left hemisphere supplementary motor area, anterior cingulate cortex, and pars opercularis (Fig. 3a; see Supplementary Table S1). Bilateral globus pallidus activation was also detected in the control group, but did not survive an extent threshold of 10 voxels (Supplementary Table S1). A-II showed left inferior frontal gyrus activity for the reverse contrast (Baseline vs. Nonword repetition, peak coordinate −58, 10, 18, T = 2.99, p = 0.002, 39 voxels; see Fig. 3b), and superior temporal activation for the Task vs. Baseline contrast at uncorrected threshold (Fig. 3c). No difference between A-II and the control group however survived correction for multiple comparisons (p = 0.05 FWE).

Two control participants also showed no detectable activation at p = 0.005, and one showed activation only in the left superior temporal gyrus during the Task vs. Baseline contrast. None of these participants showed activation in the pars opercularis for the reverse contrast.

## Discussion

Substantial bilateral structural abnormalities in the basal ganglia and hippocampus were detected in an 8 year old boy with the characteristic *FOXP2* mutation speech phenotype, providing key insights into the structural correlates of this disease. Brain cortical activity during nonsense word repetition was abnormal. These findings only partly overlap with those of affected members of the KE family, and provide us with early neural markers of *FOXP2* dysfunction.

### Basal ganglia and thalamus reductions

Both volumetric and VBM results supported our hypothesis of a reduction in the caudate nucleus, a key neural marker in the adult affected KE members^2^,^8^,^11^. Structural reductions^15^,^16^ and functional^17^ anomalies in that region have also been reported in children with language impairment^18^, indicating a likely link between caudate anomalies and language difficulties. Such a link would be consistent with caudate connectivity with frontal associative, rather than motor, circuits^19^,^20^.

Thalamic reductions were detected only with our volumetric approach. Dorsal thalamic reductions had been observed in both affected and unaffected KE members using VBM^11^, casting doubt on their relevance to the behavioural phenotype. To our knowledge, the thalamus has not been reported as abnormal in children with speech sound disorders or idiopathic childhood apraxia of speech^18^, and this is therefore a novel finding. As this reduction was only detected using volumetric analysis and did not survive correction for multiple comparisons, replication will be necessary to establish an association between *FOXP2* disruption and thalamic changes.

The largest volumetric reductions detected were located in the globus pallidus. Structural anomalies in that region have not been detected using VBM in the affected KE adults or in A-II, but it should be noted that segmentation of this region is problematic due to its low contrast on T1-weighted images^21^. Inconsistent pallidum results have been reported in speech and language impaired individuals. Pallidum enlargement has even been reported in young adults with developmental language impairment^15^ and increased fMRI activity has been reported in one study of children with speech sound error (not apraxia of speech)^22^. Conversely, reduced fMRI activity in the globus pallidus was reported in the affected KE members during a covert language task^9^. The globus pallidus (internal segment) is a major output structure of the striatum, via the thalamus to the cortex. In adulthood, the globus pallidus and thalamus are closely linked during speech tasks via subcortico-cortical networks involving the supplementary motor area, insula, premotor cortex, and inferior frontal gyrus^23^. It is therefore plausible that globus pallidus anomaly in childhood would be associated with functional and structural anomalies in these target cortical regions of the speech network later in life.

Altogether, our structural MRI findings point to early abnormal development of the basal ganglia in individuals with *FOXP2* dysfunction, consistent with a subset of the proposed original anatomical model of *FOXP2* based on expression and neuroimaging studies^3^. The early role of the basal ganglia in speech and language function is also supported both by recent language models^23^,^24^, and by the procedural learning hypothesis for language disorders^25^,^26^.

### Hippocampal reductions

Bilateral reductions of the hippocampus and surrounded cortices were unexpected and must be interpreted cautiously as only the left peak survived correction for multiple comparisons. No evidence of expression of *FOXP2/foxp2* in the mature or developing hippocampus has been previously reported in rodent or human embryo studies^27^,^28^ or in the developing monkey brain^29^. Paradoxically, enlargement of the hippocampus has been reported in young adults with language impairment^15^. The early role of the hippocampus in language development is unclear, as hippocampal function is traditionally linked to episodic memory skills, which were not tested in A-II. In principle, hippocampal reduction could limit the potential for verbal memory and language development. Recent evidence suggests that declarative memory is a significant predictor of grammar comprehension in children with language impairments, suggesting a compensatory role for this system^30^. In addition, a recent meta-analysis suggested deficits in verbal declarative memory in individuals with specific language impairment^31^. The hippocampal reductions reported here could therefore relate to the language phenotype in A-II. They could also however be coincidental, or be an indirect result of gene-environment interactions. The interaction between declarative and procedural memory circuits in developmental communication disorders warrants further investigation, and our findings point to a potential disruption of both in this boy with *FOXP2* deletion. However, it should be noted that this view of completely separate memory systems has been recently challenged^32^ by studies of patients with Parkinson’s disease^33^,^34^ and of children with language impairment^30^.

### fMRI findings

Despite performing the task aloud, A-II showed little fMRI activation during nonsense word repetition, and mirrors the findings in the KE family members^10^. It is intriguing that the peak coordinates of de-activation (negative response) in pars opercularis (−58, 10, 10) were so close to the peak coordinates of underactivation reported in the affected members of the KE family^9^ during verb generation (−60 12 12). Altogether, we have preliminary evidence that a further marker of *FOXP2*-related dysfunction could be malfunction in the inferior frontal gyrus, pars opercularis, a region functionally connected to the basal ganglia circuit via the globus pallidus^23^.

Cross-sectional neuroimaging studies do not allow us to distinguish the brain changes directly caused by *FOXP2* mutation and those indirectly affected following interactions with the environment, surrounding genes, and functional reorganization of interacting neural circuits. Based on expression studies and gene function studies^3^, our current interpretation is that caudate, thalamic and pallidum changes are a direct result of *FOXP2* anomaly. Either their structural development, their functional development (via dopaminergic systems), or both, are disrupted. Hippocampus reductions could, by contrast, be the result of behavioural compensation or neural reorganization of circuits, as a result of altered interaction with the environment.

In conclusion, our findings suggest that large subcortical reductions are strong markers of *FOXP2* disruption in childhood. A combination of bilateral reduction in the caudate nucleus, globus pallidus, and hippocampus may therefore also be a promising biomarker of severe and long lasting speech and language impairments arising from of other genetic causes. These regions could therefore also serve as targets for novel therapeutic strategies.

## Methods

### Participants

#### Case A-II

A-II’s early history and current neuropsychological profile are detailed elsewhere^7^. In brief, he has a diagnosis of oral motor apraxia, severe childhood apraxia of speech, and dysarthria. Concomitant impairments in receptive and expressive language as well as literacy (<5^th^ centile on all tests) are present. Speech is highly unintelligible due to reduced consonant inventory, imprecise articulation, delayed phonological processes, and atypical error patterns. Both speech and non-speech oromotor functions are impaired (<5^th^ percentile). In contrast, nonverbal intelligence as measured using Performance IQ lies within the average range. A-II was aged 8 years 11 months at the time of scanning.

Our team recently diagnosed a submucous cleft during our research evaluations, despite A-II being seen by community speech pathologists as well as an ear nose and throat surgeon since a young age. He recently had surgery to correct this anomaly, with resultant improvement in speech resonance.

#### Control groups

A-II’s MRI data were compared to those of 24 typically developing term-born males aged 8 to 11 years recruited via a community prospective longitudinal study^35^ in metropolitan Melbourne, Australia. These control participants had been followed up between 8 months and 7 years and had never had a diagnosis of hearing deficit, speech or language impairment or delay, neurological symptoms, genetic disorder, or pervasive developmental disorder. Two additional 8-year-old males were recruited via advertisement to widen the age range, using the same exclusion criteria. All 26 control participants were native monolingual English speakers.

Some datasets were either missing or unusable due to excessive motion artefact, and therefore the final number of control participants differs slightly between VBM (N = 26, mean age = 10 years 3 months, SD = 8.3 months, no data excluded) and fMRI (N = 25, mean age = 10 years 2 months) analyses. Age ranged from 8 years 3 months to 11 years 2 months in both cases. As tractography was performed in native space, only a subgroup of males closest in age to A-II was investigated (N = 14 after one dataset was excluded due to excessive motion artefact; mean age 9 years 9 months, SD = 5.5 months, range 8 years 3 months to 10 years 5 months).

### MRI acquisition

All MRI data were acquired on a 3T Siemens Skyra scanner (Erlangen, Germany).

#### Structural MRI

3D T1-weighted Magnetization Prepared Rapid Gradient Echo (MPRAGE) images were acquired with 0.9 mm isotropic resolution (TR: 1900 ms; TE: 2.49 ms; flip angle: 9°; matrix size: 256 × 256; FoV: 240 mm).

#### Diffusion MRI

Diffusion data were acquired with 64 diffusion directions at b = 3000 s/mm^2^ and 1 b = 0 s/mm^2^, voxel size = 2.5 mm isotropic, and TR/TE = 6800/110 ms.

#### Functional MRI

All functional images were acquired aligned along the AC-PC plane using an echo planar imaging (EPI) sequence with whole-brain coverage (TR: 3000 ms; TE: 30 ms; flip angle: 90°; 44 interleaved slices; matrix size: 72 × 72; 3 mm isotropic voxels; FoV: 216 mm).

#### FMRI nonword repetition task

We used a block design paradigm alternating five blocks of Baseline and five blocks of Task (12 s duration each). The order of presentation was counterbalanced across the group, with 12 participants starting with Baseline, and 14 (including A-II) starting with Task. Participants were instructed to look at a cross presented on a screen throughout each functional MRI scan. During the Task period (“nonword repetition”), participants were required to repeat 35 nonsense words aloud (seven per block) presented via inner ear buds. Participants were given 3 seconds (ISI–5 sec) to provide an overt response to the nonword stimuli. Nonwords were two to five syllables long, with 28 from the Children’s Nonword repetition Test^36^ and seven from the Nonword Memory test (with permission from the author https://www.york.ac.uk/res/wml/test%20of%20WM.html). During the Baseline period, participants were asked to listen to frequency and duration matched bursts of pink noise (“Pink noise”) generated using the Praat software (www.fon.hum.uva.nl/praat/). Participants practiced the nonword repetition task and completed a mock scan prior to the actual MRI scan. Overt responses were recorded using Audacity software (*audacity.sourceforge.net/*) via a custom made noise cancelling microphone. This procedure ensured participants were completing the task and allowed for offline scoring of responses.

### MRI analysis

#### VBM

We used VBM^37^ to identify grey matter differences at the whole-brain level (as used in previous KE family studies^8^). The results informed the choice of an additional region of interest (the hippocampus) for regional volumetric analyses. T1-weighted datasets were analysed within SPM12 (http://www.fil.ion.ucl.ac.uk/spm/software/spm12). Firstly, data were segmented into grey matter, white matter and cerebrospinal fluid using DARTEL and a custom template generated from all participants. Segmented data were modulated then smoothed using a large smoothing kernel (12 mm FWHM), as recommended for unbalanced designs^38^ and in particular for single subject analyses. Focal regions of increased and decreased grey matter in A-II were identified using analysis of covariance (ANCOVA) with age and total grey matter volumes as covariates. Equality of variance was assumed, as recommended for single case designs^39^.

Differences significant at p = 0.05 corrected for family-wise error were reported. Voxels significant at p < 0.001, uncorrected for multiple comparisons were also reported where we had a-priori hypotheses, that is, in the basal ganglia and inferior frontal gyrus^11^. Small volume correction was used in the caudate nucleus and putamen where we had an *a priori* hypothesis, using the images generated from volumetric analyses (see below) as masks.

Of note, given our single case design and large smoothing kernel (12 mm, see Methods) for VBM, our methods had reduced sensitivity to detect differences at smaller spatial scales than 12 mm, as is likely to be the case in cortical and cerebellar regions.

#### Volume extraction in regions of interest

Whole-brain grey and white matter volumes were extracted using SPM12. Volumes from the basal ganglia (a-priori hypothesized) and hippocampus (based on VBM findings) were extracted using FSL FIRST^40^ (http://fsl.fmrib.ox.ac.uk/fsl/fslwiki/FIRST) which uses automatic subcortical segmentation. Volumes were subsequently adjusted for total grey matter. Segmentation errors were checked in all cases, and manually corrected using published protocols^41^,^42^.

Since VBM and direct volumetric analyses are subject to different methodological sensitivities, they may not necessarily result in identical findings. For example, while VBM does reflect absolute volume changes when using modulated data as in the present case, the segmentation of the grey matter structures of interest are achieved by quite different processes. In addition, the fact that VBM is a voxel based method (i.e. statistical tests are performed on a voxel by voxel basis), the requirement for multiple comparison correction is very different in the two approaches, and this is compounded by the above-mentioned need to use a large smoothing kernel for single subject VBM. Nevertheless, there is evidence that the two methods produce similar findings, particularly in subcortical structures in healthy individuals^43^, and they are therefore regarded as providing complementary information.

#### Diffusion Weighted Imaging (DWI) tractography

Given the disadvantages of the diffusion tensor model and of deterministic tractography methods for reconstructing fibre tracts^44^, here we used DWI probabilistic tracking based on constrained spherical deconvolution (CSD using MRtrix^45^ version 0.2 software). This method has been shown to be particularly advantageous in regions of crossing fibers^46^. We also used MRtrix to calculate the diffusion tensor and to derive the fractional anisotropy (FA) in all voxels. FA analysis was performed in voxels within tract regions of interest identified from fibre tracking. We examined the FA characteristics of essential speech and language-related pathways, namely (i) the corticobulbar tract (originating in the ventral part of the motor cortex, corresponding to the lips/larynx/tongue representations) and (ii) the direct and dorsal segments of the arcuate fasciculus. The left and right cingulum bundles and corticospinal tracts (originating from the hand region) were used as control tracks, as they were not hypothesized to be related to the phenotype. We used tractography methods previously validated in children with other neurological conditions^47^,^48^ (see Supplementary Methods).

### fMRI

Individual data were realigned, coregistered, normalized to the MNI template, and smoothed using an 8 mm full width at half maximum kernel within SPM8 using the default parameters. First level analysis involved the comparison of Task (Nonword repetition) vs. Baseline contrasts using motion parameters generated from the realignment process as regressors. Second level analysis involved the comparison of Case-A-II vs. control group in an ANCOVA, with age as covariate of no interest. Equality of variance was assumed.

Given previous report of altered activation in the affected members of the KE family in Broca’s area but not in the superior temporal gyrus^9^, mean signal change and time series data were extracted from peak voxel within these two regions of interest using the “plot” function within SPM8.

#### Statistical analysis for volumetric and tractography-derived data

All numerical data were transformed into z-scores calculated using the mean and standard deviations of the control group as recommended for case-control designs. We reported the p value based on a one-tailed one-sample t-test, and provided confidence interval of the effect size and percentiles scores^49^ calculated using a customized program (www.abdn.ac.uk/~psy086/dept/Single_Case_Effect_Sizes.htm). This calculation assumes non-central t distribution of the control data rather than normal distribution, and therefore takes sample size into account. We estimated that normal distribution was not appropriate in our study as our control sample sizes were moderate, and therefore the risk of Type I error was inflated.

#### Statistical analysis for whole-brain MRI comparisons

VBM analyses comparing a single participant to a control group can result in false positives. To overcome this, we employed a similar method to that described in Muhlau *et al.*^39^ whereby each individual dataset was compared to that of the rest of the control group. For the fMRI analysis, each control’s activation map was examined individually.

### Ethics

The study was approved by the Royal Children’s Hospital Human Research Ethics Committee (HREC 27053 and #31225) and carried out in accordance with the provisions of the World Medical Association Declaration of Helsinki. All parents/guardians gave informed consent. Families were reimbursed for travel expenses.

## Additional Information

**How to cite this article**: Liegeois, F. J. *et al.* Early neuroimaging markers of *FOXP2* intragenic deletion. *Sci. Rep.* **6**, 35192; doi: 10.1038/srep35192 (2016).

## Supplementary Material

Supplementary Information

## Figures and Tables

**Table 1:** Relative volumes (in percentage of total grey matter) in regions of interest in A-II and in the control group (N = 26).

Region of interest		Control group mean (SD)	A-II’s value	Estimated percentile	Significance test (one tailed)	Estimated effect size for difference (95% CI)
Hippocampus	Left	0.471 (0.047)	0.376	3.63%	t = −1.953; p = 0.036	−2.021 (−2.939 to −1.079)
	Right	0.473 (0.046)	0.420	14.29%	t = −1.113; p = 0.142	−1.152 (−1.822 to −0.456)
Caudate nucleus	Left	0.471 (0.056)	0.350	2.85%	t = −2.087; p = 0.029	−2.161 (−3.123 to −1.175)
	Right	0.481 (0.054)	0.354	2.04%	t = −2.272; p = 0.020	−2.352 (−3.376 to −1.305)
Globus pallidus	Left	0.210 (0.018)	0.155	0.56%	t = −2.952; p = 0.0056	−3.056 (−4.319 to −1.773)
	Right	0.210 (0.015)	0.183	5.28%	t = −1.739; p = 0.053	−1.800 (−2.649 to −0.926)
Thalamus	Left	1.049 (0.068)	0.919	4.38%	t = −1.847; p = 0.044	−1.912 (−2.795 to −1.004)
	Right	1.025 (0.067)	0.895	4.17%	t = −1.875; p = 0.042	−1.940 (−2.832 to −1.023)
Putamen	Left	0.624 (0.068)	0.580	27.14%	t = −0.625; p = 0.27	−0.647 (−1.216 to −0.058)
	Right	0.600 (0.060)	0.543	18.77%	t = −0.918; p = 0.19	−0.950 (−1.574 to −0.301)

**Table 2:** Mean FA measures derived from tractography in A-II and in the control group.

Track		Control group mean (SD)	A-II’s value	Estimated percentile	Significance test (one tailed)	Estimated effect size for difference (95% CI)
Corticospinal tract	Left	0.393 (0.0146)	0.387	17.67%	t = −0.377; p = 0.35	−0.390 (−0.928 to 0.161)
	Right	0.399 (0.0153)	0.396	43.60%	t = −0.164; p = 0.43	−0.170 (−0.695 to 0.361)
Corticobulbar tract	Left dorsal	0.445 (0.0705)	0.3874	22.09%	t = −0.793; p = 0.22	−0.821 (−1.419 to −0.200)
	Right dorsal	0.447 (0.0566)	0.3614	8.37%	t = −1.462; p = 0.084	**−1.513** (−2.279 to −0.722)
	Left ventral	0.396 (0.0475)	0.3874	43.19	t = −0.175; p = 0.43	−0.181 (−0.706 to 0.350)
	Right ventral	0.422 (0.0635)	0.3745	24.32%	t = −0.716; p = 0.24	−0.741 (−1.325 to −0.136)
Arcuate fasciculus	Left	0.437 (0.0262)	0.4027	11.31%	t = −1.270; p = 0.11	**−1.315** (−2.026 to −0.578)
	Right	0.404 (0.0277)	0.4349	66.19%	t = 1.064; p = 0.15	**1.101** (0.418 to 1.759)
Dorsal (SLF) portion of arcuate	Left	0.419 (0.0212)	0.395	14.44%	t = −1.106; p = 0.14	**−1.145** (−1.813 to −0.451)
	Right	0.400 (0.0188)	0.4185	82.05%	t = 0.947; p = 0.18	0.980 (0.325 to 1.611)
Cingulum bundle	Left	0.348 (0.0241)	0.3102	7.53%	t = −1.527; p = 0.075	**−1.581** (−2.365 to −0.771)
	Right	0.335 (0.0299)	0.3444	61.92%	t = 0.310; p = 0.38082	0.321 (−0.223 to 0.853)

Z values that fall one standard deviation below and above the control mean are highlighted in bold.
