# Supplementary material for: Early neuroimaging markers of FOXP2 intragenic deletion
Source: Sci Rep. 2016 Oct 13;6:35192. doi: 10.1038/srep35192 (PMC5062117; doi:10.1038/srep35192)
Supplement: Supplementary Information [file srep35192-s1.pdf]

Early neuroimaging markers of FOXP2 intragenic deletion

Frederique J Liegeois*[1,2], Michael Hildebrand[3,4,5], Alexandra Bonthrone[1,2], Samantha J Turner[3,6], Ingrid E Scheffer[3,4,5,8], Melanie Bahlo[3,8], Alan Connelly[3,4], Angela T. Morgan[3,6,7]

1. UCL Institute of Child Health, London, UK
2. Great Ormond Street Hospital for Children NHS Trust, London, UK
3. University of Melbourne, Australia
4. Florey Institute of Neuroscience and Mental Health, Melbourne, Australia
5. Austin Health, Melbourne, Australia
6. Murdoch Childrens Research Institute, Melbourne, Australia.
7. Royal Children's Hospital, Melbourne, Australia
8. The Walter and Eliza Hall Institute of Medical Research, Melbourne, Australia

***Corresponding author:** Frédérique Liégeois. Cognitive Neuroscience and Neuropsychiatry Section, UCL Institute of Child Health, 30 Guilford Street, London, UK, WC1N 1EH. Tel: +44 (0)20 7905 2728 Fax: +44(0)207 905 2616. E-mail: f.liegeois@ucl.ac.uk

Supplementary RESULTS

Supplementary Table S1. Control group (N=25) activation for the Nonword repetition vs. Baseline contrast (extent threshold 10 voxels). Only peaks surviving a threshold of p=0.05 with family-wise error (FWE) correction are reported. Globus pallidus activation is reported for information even though cluster size is below 10 voxels as it was a-priori hypothesized.

**Anatomical region**	**Peak coordinates (x, y, z)**	**T value**	**Cluster size** (voxel count)	**P value** (peak level, FWE corrected)
Right pre/post central gyrus	42, -12, 38	12.02	264	<0.001
Right pre/post central gyrus	-44, -18, 42	10.83	260	<0.001
Right posterior superior temporal gyrus extending anteriorly	52, -20, 0	8.27	210	<0.001
Left supplementary motor area	-4, 8, 60	7.52	53	0.001
Right anterior superior temporal gyrus	40, 12, -18	6.78	84	0.005
Left superior temporal gyrus	-58, -14, -2	6.60	24	0.007
Left anterior cingulate	-6, 14, 38	6.09	26	0.019
Left pars opercularis	-56, -4, 22	5.96	17	0.024
Left globus pallidus	-22, -14, 16	5.78	4	0.034
Right globus pallidus	18, -10, 16	5.83	8	0.023

Supplementary METHODS

Tractography

All seed regions were manually drawn using the MRtrix ROI tool on colour coded tensor eigenvector maps in native space. In brief, the long segment of the arcuate fasciculus was tracked using a seed region in the "bottleneck" of the arcuate/superior longitudinal fasciculus (three consecutive slices in coronal plane), with a target region in the bend (two contiguous slices in axial plane)[48]. When necessary, exclusion regions were placed in the corpus callosum (whole cross section at midline level, in sagittal plane). The direct segment of the arcuate was tracked using the same bottleneck seed region as for the long segment, with a second inclusion region placed more anteriorly in the frontal lobe in coronal plane (two contiguous slices), and a large exclusion region in the axial plane to exclude streamlines that enter the temporal lobe. The cingulum bundles were tracked using an anterior seed region perpendicular to the bundle (two contiguous slices in coronal plane) and an inclusion region in the posterior part (two contiguous slices in coronal plane). The whole corpus callosum cross section was used as exclusion region to avoid capturing interhemispheric tracts.

A threshold was then applied to each generated track to exclude voxels containing 40 streamlines or less, and a binary mask containing retained voxels was generated. A threshold of 10 streamlines was used for corticobulbar or corticospinal tracks as tractography reconstruction showed very few spurious streamlines. Finally, mean volume (number of voxels within the binary mask) and mean FA (averaged across all voxels within the binary mask) were extracted for each participants and each track within MRtrix. All 180 tracks were successfully extracted.

Supplementary DISCUSSION

Weak white matter anomalies in the dorsal stream

To our knowledge, we present the first tractography study of individuals with *FOXP2* disruption. FA reductions in speech and language white matter pathways were not statistically significant, with effect sizes approximating one standard deviation in the dorsal white matter tracts. Two recent studies have reported FA reductions in the superior longitudinal fasciculus[50] and other dorsal and ventral language related pathways[51] for children with idiopathic language impairment. Our single case design may however not have provided the necessary power to detect these differences. A longitudinal follow-up of A-II would allow us to examine whether the borderline white matter anomalies identified here resolve, or on the contrary become more severe with increasing age.

Supplementary References

50 Verhoeven, J. S. *et al.* Is there a common neuroanatomical substrate of language deficit between autism spectrum disorder and specific language impairment? *Cereb Cortex* **22**, 2263-2271, doi:10.1093/cercor/bhr292 (2012).

51 Vydrova, R. *et al.* Structural alterations of the language connectome in children with specific language impairment. *Brain and language* **151**, 35-41, doi:10.1016/j.bandl.2015.10.003 (2015).
